# In silico and phylogenetic analyses of partial *BbRAP-1*, *BbCP2*, *BbSBP-4* and *BbβTUB* gene sequences of *Babesia bovis* isolates from cattle in South Africa

**DOI:** 10.1186/s12917-017-1261-7

**Published:** 2017-12-08

**Authors:** Phillip Senzo Mtshali, Moses Sibusiso Mtshali

**Affiliations:** 1 Veterinary Parasitology Programme, Research and Scientific Services Department, National Zoological Gardens of South Africa, Pretoria, 0001 South Africa; 2 Parasitology Research Programme, Department of Zoology and Entomology, University of the Free State, QwaQwa Campus, Phuthaditjhaba, 9866 South Africa

**Keywords:** *Babesia bovis*, Cattle, South Africa, Nested PCR assays, Phylogenies

## Abstract

**Background:**

Bovine babesiosis is one of the most economically important tick-borne diseases threatening the livestock industry globally including South Africa. This disease is induced by members of *Babesia bovis* species. Antigenic variations among geographical strains of *B. bovis*, and these heterogeneities are cited as the mechanism by which parasites evade from host immune system and they hamper the successful development of a single vaccine that could confer absolute protection. Given the economic importance of livestock industry in South Africa, the extent of genetic diversity among field isolates of *B. bovis* merits extensive investigation.

In this study, we genetically characterized partial genes of *B. bovis* and studied the phylogenetic relationship among *B. bovis* isolates of South African origin. The genes, which were PCR-amplified from bovine samples collected from different locations across South Africa, coded for rhoptry-associated protein 1 (*BbRAP-1*), cysteine peptidase 2 (*BbCP2*), spherical body protein 4 (*BbSBP-4*) and β-tubulin (*BbβTUB*). Phylogenies were inferred from newly determined sequences using the neighbour-joining approach.

**Results:**

Nested PCR assays with gene-specific primers indicated that, of the 54 bovine samples tested, 59.3% (32/54; 95% CI = 46.0–71.3%), 27.8% (15/54; 95% CI = 17.6–40.9%), 37.0% (20/54; 95% CI = 25.4–50.4%) and 29.6% (16/54; 95% CI = 19.1–42.8%) possessed *BbRAP-1*, *BbCP2*, *BbSBP-4* and *BbβTUB* fragments, respectively. Sequencing of PCR-generated fragments revealed that nucleotide sequences of each of the four genes were highly conserved among the *B. bovis* isolates examined. Phylogenetic analyses of *BbCP2*, *BbSBP-4* and *BbβTUB* sequences indicated a close phylogenetic relatedness among South African-derived sequences and those of global *B. bovis* strains.

**Conclusion:**

The data reported in this study indicated that there is a high conservation among the genes of *B. bovis* isolates from cattle in South Africa. These findings give an indication that immunologically important proteins encoded by these genes could potentially be considered for exploitation as viable candidates for inclusion in recombinant subunit vaccines.

## Background

*Babesia bovis* is an intraerythrocytic protozoan parasite and an important aetiological agent of bovine babesiosis. This tick-borne disease is considered an economically important constraint to livestock production in tropical and subtropical regions of the world [1]. While *B. bigemina* (another agent of babesiosis) can be transmitted by both *Rhipicephalus microplus* and *R. decoloratus*, the confirmed tick vector of *B. bovis* in southern Africa is *R. microplus* [2].

Cattle infected by *B. bovis* are characterized by high fever, ataxia, anorexia and sometimes nervous signs [3]. Further, *B. bovis* remains the most pathogenic microorganism compared to other *Babesia* species and induces a more severe disease due to the sequestration of infected red blood cells in cerebral capillaries, thus resulting in parasitaemia levels of less than 1% in the circulating blood [4, 5]. On the other hand, the disease caused by *B. bigemina* is milder and is characterized by anaemia, fever and haemoglobinuria. Infections by *B. bigemina* do not involve sequestration of infected red blood cells and the levels of parasitaemia in blood often exceed 10% [3]. Therefore, it remains pivotal that effective control measures are implemented in an endeavour to curtail cattle mortalities resulting from *Babesia* infections.

In South Africa, vaccination against *B. bovis* is a method that is widely exploited to immunize cattle against babesiosis. This follows that the cattle which recover from primary acute infection remain persistently infected and serve as reservoirs for parasite transmission to other animals [4]. Nevertheless, it is worth noting that the available vaccines are based on bovine blood infected with live attenuated strains of the parasite [6, 7]. Although these vaccines confer protective immunity, the major limitation associated with the use of attenuated vaccines relate to the possible transmission of other blood-borne pathogens [6]. In addition, the production of live vaccines requires artificial infection of cattle in order to attain high parasitaemia levels in the blood, and this often constitutes important ethical implications [8].

According to Torina et al. [9], the success in the development of vaccines against babesial infections is impeded by the presence of heterogeneities in some parasite proteins. In particular, studies are focused on surface proteins that can confer high level of protection and better safety in comparison to currently used vaccines [10]. Many of these proteins might play a crucial role in erythrocyte invasion and are therefore the targets for vaccine development [9]. Several genes encoding these *B. bovis* proteins with immunogenic potential are known, and thanks to the complete genome sequencing of an American *B. bovis* T2Bo strain that these surface proteins were identified [11]. However, it must be acknowledged that there is still a dearth of information regarding the conservation of genes encoding potential immunogenic proteins in South African isolates of *B. bovis*.

In this context, the present study was borne out of the need to characterize the genes encoding rhoptry-associated protein 1 (*BbRAP-1*), cysteine peptidase 2 (*BbCP2*), spherical body protein 4 (*BbSBP-4*) and β-tubulin (*BbβTUB*) in *B. bovis* isolates from South African field bovine samples.

## Methods

### Blood samples

Field bovine samples (*n* = 54) were randomly selected to validate the nested PCR assays developed in this study. These samples form part of the sample collection of the Veterinary Parasitology Programme (National Zoological Gardens of South Africa: NZG, South Africa), and the collection of these samples was approved by the NZG Research Ethics and Scientific Committee. Animal owners gave verbal informed consent for the collection of the samples for the epidemiological survey, and animals were not involved in any clinical trials or treatments. All blood samples were maintained at −20°C prior to DNA extraction.

### DNA extraction

Genomic DNA was extracted using ZR Genomic DNA™-Tissue MiniPrep kit (Zymo Research Corporation, Irvine, CA, USA) according to the manufacturer’s instructions. DNA quantification was performed using a NanoDrop^®^ ND-1000 (NanoDrop Technologies Inc., Wilmington, USA).

### Primer design

Nested PCR primers used for amplifying *B. bovis*-specific *BbRAP-1* genes from bovine samples were reported previously [12]. Primers targeting *BbCP2*, *BbSBP-4* and *BbβTUB* genes were created with Primer-BLAST program of the NCBI using reference sequences BBOV_IV007730 (GenBank accession number XM_001610645), BBOV_IV005390 (XM_001610418) and BBOV_III004850 (XM_001611566), respectively. All primers shown in Table 1 were synthesized by Inqaba Biotechnical Industries (Pretoria, South Africa).

**Table 1:** Primer sets used to amplify DNA fragments specific for *Babesia bovis*

Gene	Assay	Primer name	Oligonucleotide primers (5′ → 3′)	Annealing	Product size^a^	Reference
*BbRAP-1*	PCR	BoF	F-CACGAGGAAGGAACTACCGATGTTGA	55 °C	360 bp	[12]
		BoR	R-CCAAGGAGCTTCAACGTACGAGGTCA			[12]
	nPCR	BoFN	F-TCAACAAGGTACTCTATATGGCTACC	57 °C	298 bp	[12]
		BoRN	R-CTACCGAGCAGAACCTTCTTCACCAT			[12]
*BbCP2*	PCR	CpBovF	F-TGCATCGGACCTATCCAACC	57 °C	960 bp	This study
		CpBovR	R-TCAGCAGCCAAATAAGGCCA			This study
	nPCR	CpBov3	F-ATCGGAAGAAGTCGCCGTTG	65 °C	829 bp	This study
		CpBov4	R-AAGCGTAGTCGCTGTAACCA			This study
*BbSBP-4*	PCR	BbSBP1	F-AGTTGTTGGAGGAGGCTAAT	57 °C	887–905 bp	This study
		BbSBP2	R-CTTCTCGGCGTCCTTTTC			This study
	nPCR	BbSBP3	F-CCGCATTCTTAAGACTTCTGA	60 °C	726–744 bp	This study
		BbSBP4	R-GTTACCATTTCATCGTTGTCA			This study
*BbβTUB*	PCR	BTbovA	F-AGAGCGGTACTTACCACGGA	61 °C	1203 bp	This study
		BTbovB	R-CGTCGTCGATGGTTGCTTCT			This study
	nPCR	BTbovC	F-GTTCCACGCGCTGTACTCAT	65 °C	954 bp	This study
		BTbovD	R-CATGTCCTGGATGGCGGTAG			This study

^a^Theoretical product sizes based on nucleotide gene sequences of several *B. bovis* strains used as templates for primer design

### Amplification of genes

PCR was performed in a 25-μl reaction containing 2.5 μl of the extracted DNA template, 0.6 μM of each primer and 12.5 μl of 2× DreamTaq Green PCR Master Mix (Inqaba Biotechnical Industries). The reactions were subjected to thermal conditions in a Bio-Rad T100™ thermal cycler (Bio-Rad Laboratories, Johannesburg, South Africa) with the following temperature profiles: 94 °C for 3 min, followed by 35 cycles of 94°C for 30 s, 55–65 °C for 45 s and 72 °C for 1 min. This was followed by a final extension at 72 °C for 10 min. The specific annealing temperatures are shown in Table 1. For nested PCR, 1 μl of the amplified PCR products was used as a template. Nested PCR products were subjected to gel electrophoresis in 1.2% (*w*/*v*) agarose gels stained with Biotium GelRed Acid Stain (Anatech Instruments, Johannesburg, South Africa) and visualized under ultraviolet illumination. GeneRuler 100-bp DNA Ladder (Inqaba Biotechnical Industries) was used as the standard molecular weight marker.

### DNA sequencing and phylogenetic analysis

Nested PCR products of positive samples, and whose GenBank accession numbers appear in Table 2, were selected for subsequent sequencing of *BbCP2*, *BbSBP-4* and *BbβTUB* genes. To corroborate correct amplification of *BbRAP-1* genes, 12 positive samples were sequenced. Nucleotide sequences were determined with ABI 3130XL Genetic Analyzer (Applied Biosystems, Johannesburg, South Africa) using a Big Dye Terminator Kit (Applied Biosystems). For each gene, DNA sequencing was performed on both strands using the corresponding forward and reverse primers. Multiple sequence alignments were performed using a Biological Sequence Alignment Editor [13]. Neighbour-joining trees were created using MEGA v5.0 software [14]. Molecular distances were estimated with Kimura two-parameter model [15], and the robustness of branches was determined using bootstrapping analysis with 1000 replicates [16]. Pairwise comparisons of nucleotide sequences were performed with EMBOSS Needle program (version 6.3.1) of the European Bioinformatics Institute (http://www.ebi.ac.uk/tools/psa/emboss_needle). Nucleotide sequences were translated to protein sequences using ExPASy translate tool (http://web.expasy.org/translate).

**Table 2:** GenBank accession numbers of nucleotide sequences generated in this study

Province	Sample ID	GenBank accession number		
		*BbCP2*	*BbSBP-4*	*BbβTUB*
KwaZulu-Natal	KZN-C1	–	KF626629	–
	KZN-C2	KF626619	KF626630	KF626639
	INDIA	KF626620	KF626631	KF626640
Mpumalanga	MP-C16	KF626621	–	–
	MP-C17	–	–	KF626641
	MP-C18	KF626622	KF626632	KF626642
Western Cape	WC-10270	KF626623	KF626633	KF626643
Gauteng	GP-C7	KF626624	KF626634	KF626644
	GP-C15	–	KF626635	–
	GP-C17	KF626625	KF626636	KF626645
Eastern Cape	EC-23A	KF626626	–	–
North West	NW-C2	KF626627	KF626637	KF626646
	NW-C4	KF626628	KF626638	KF626647

(−-) No sequences generated

### Nucleotide sequence accession numbers

Nucleotide sequences determined in this study were deposited in GenBank under the accession numbers KF626619 – KF626647.

## Results

### Amplification of target genes by PCR

The nested PCR primers specifically designed to detect *B. bovis* DNA in bovine samples yielded single amplicons of 298 bp for *BbRAP-1*, 829 bp for *BbCP2*, 726–744 bp for *BbSBP-4* and 954 bp for *BbβTUB*. Nested PCR assays with primers targeting four different genes were able to detect *B. bovis* DNA from some of the tested bovine samples. For example, BoFN/BoRN primers detected the *BbRAP-1* genes in 32 out of 54 samples tested (59.3%). On the other hand, a total of 15 (27.8%), 20 (37.0%) and 16 (29.6%) samples possessed *BbCP2*, *BbSBP-4* and *BbβTUB* fragments, respectively. In testing the specificity of PCR and nested PCR assays, purified DNA samples of *B. bigemina*, *Anaplasma centrale*, *Ehrlichia ruminantium* and *Theileria parva* were employed as negative controls, and as such, they yielded no PCR amplifications.

In order to corroborate if the correct PCR fragments were amplified, 12 samples possessing the *BbRAP-1* gene and representing seven provinces were sequenced. The resulting nucleotide sequences were subjected to homology searches in GenBank. The *BbRAP-1* sequences determined in this study exhibited 99–100% identity with published sequences of *B. bovis* strains originating from Philippines (JX860283), Brazil (FJ588009 to FJ588013 and AF030057 to AF030058), Uruguay (AF030060 to AF030061), Argentina (AF030053 and AF030056), USA (AF030054 and AF030059) and Cuba (JF279443).

### In silico analysis of *B. bovis* sequences

Among the samples showing positive PCR amplifications, samples whose GenBank accession numbers appear in Table 2 were selected for sequencing. Alignment of 10 *BbCP2* gene sequences determined in this study revealed the existence of single nucleotide polymorphisms (SNPs) between the sequences. The sequences from NW-C4, GP-C7, GP-C17 and MP-C16 shared 100% identity, and contained 5 nucleotide differences in relation to MP-C18 sequence. Similarly, the sequences from EC-23A, NW-C2 and WC-10270 shared 100% identity, while the highest identity recorded with sequences from KZN-C2 and INDIA was 98.9% (9 nucleotide differences). A *blastn* search in GenBank exhibited that the sequences determined in this study were most closely related to *B. bovis* strains identified in cattle from other parts of the world. In particular, our sequences exhibited 99% identity with sequences of *B. bovis* strains R1A (GQ412131), M3P (GQ412133), S2P (GQ412136), Veracruz (GQ412135), Uruguay (GQ412134) and Brazil (GQ412132).

**Fig. 1:**
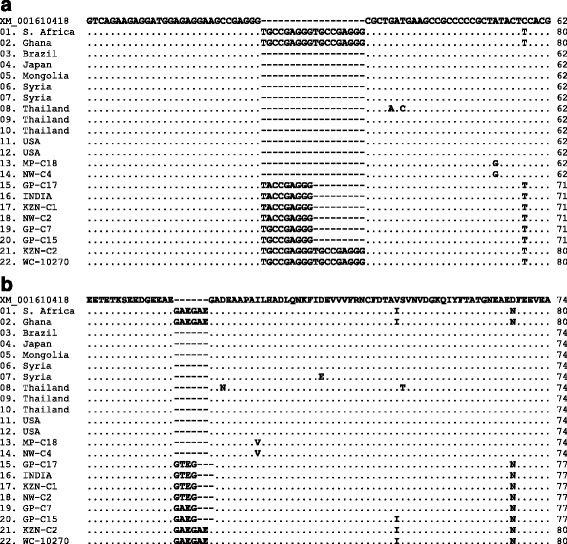
Multiple sequence alignments of partial *BbSBP-4* nucleotide gene sequences (**a**) and deduced amino acid sequences (**b**) of 12 GenBank strains and 10 South African isolates of *Babesia bovis*. The dots and dashes respectively indicate sequence identities and gaps in relation to a reference sequence (accession number XM_001610418). Accession numbers of sequences extracted from GenBank (01 to 14) correspond to those given in Table 3

Pairwise comparison of the *BbSBP-4* sequences using EMBOSS Needle program indicated a close genetic similarity between sequences of South African *B. bovis* isolates and world strains. As shown in Table 3, the *BbSBP-4* sequences from samples designated KZN-C2 and WC-10270 shared 100% identity with sequences of *B. bovis* strains previously identified in cattle from Ghana (AB569301) and South Africa (AB569303). The *BbSBP-4* sequence of GP-C17 indicated 99.9% identity with the sequence of INDIA sample and 100% identity with that of KZN-C1. In an in silico analysis of *BbSBP-4* gene sequences derived from MP-C18, NW-C4, reference strain (XM_001610418) and other world strains (Fig. 1a), it was worth noting that these sequences possessed a gap with 18 nucleotides missing in relation to sequences originating from KZN-C2, WC-10270 and two other published sequences (AB569301 and AB569303). A similar trend was observed after translating nucleotide sequences to protein sequences; the latter strains also possessed a gap with six amino acid residues missing (Fig. 1b). Similarly, the *BbSBP-4* sequences from NW-C2, GP-C7, GP-C15, GP-C17, INDIA and KZN-C1 had 9 nucleotides missing (Fig. 1a), sharing between 98.1 and 98.8% sequence identities. Despite the high percentage identities observed among closely related sequences, there were SNPs occurring at different positions across the length of *BbSBP-4* gene sequences (Fig. 1a).

**Table 3:** Pairwise comparisons of the *BbSBP-4* nucleotide gene sequences of South African *Babesia bovis* isolates and GenBank strains

Isolate origin		01	02	03	04	05	06	07	08	09	10	11	12	13	14	15	16	17	18	19	20	21	22
South Africa	01	100	100	94.5	94.5	94.2	94.4	94.4	93.7	94.4	94.5	94.5	94.5	94.8	94.8	98.3	98.1	98.3	98.1	98.3	98.8	100	100
Ghana	02		100	94.5	94.5	94.2	94.4	94.4	93.7	94.4	94.5	94.5	94.5	94.8	94.8	98.3	98.1	98.3	98.1	98.3	98.8	100	100
Brazil	03			100	100	99.7	99.9	99.9	99.2	99.9	100	100	100	98.6	98.6	96.1	95.9	96.1	96.2	96.2	95.6	94.5	94.5
Japan	04				100	99.7	99.9	99.9	99.2	99.9	100	100	100	98.6	98.6	96.1	95.9	96.1	96.2	96.2	95.6	94.5	94.5
Mongolia	05					100	99.6	99.6	99.2	99.6	99.7	99.7	99.7	98.3	98.3	95.8	95.6	95.8	95.9	95.9	95.4	94.2	94.2
Syria	06						100	99.7	99.0	99.7	99.9	99.9	99.9	98.5	98.5	95.9	95.8	95.9	96.1	96.1	95.5	94.4	94.4
Syria	07							100	99.0	99.7	99.9	99.9	99.9	98.5	98.5	95.9	95.8	95.9	96.1	96.1	95.5	94.4	94.4
Thailand	08								100	99.0	99.2	99.2	99.2	97.9	97.9	95.2	95.1	95.2	95.4	95.4	94.8	93.7	93.7
Thailand	09									100	99.9	99.9	99.9	98.5	98.5	95.9	95.8	95.9	96.1	96.1	95.5	94.4	94.4
Thailand	10										100	100	100	98.6	98.6	96.1	95.9	96.1	96.2	96.2	95.6	94.5	94.5
USA	11											100	100	98.6	98.6	96.1	95.9	96.1	96.2	96.2	95.6	94.5	94.5
USA	12												100	98.6	98.6	96.1	95.9	96.1	96.2	96.2	95.6	94.5	94.5
MP-C18	13													100	100	96.3	96.2	96.3	96.5	96.5	95.9	94.8	94.8
NW-C4	14														100	96.3	96.2	96.3	96.5	96.5	95.9	94.8	94.8
GP-C17	15															100	99.9	100	99.9	99.7	99.5	98.3	98.3
INDIA	16																100	99.9	99.7	99.6	99.3	98.1	98.1
KZN-C1	17																	100	99.9	99.7	99.5	98.3	98.3
NW-C2	18																		100	99.9	99.3	98.1	98.1
GP-C7	19																			100	99.5	98.3	98.3
GP-C15	20																				100	98.8	98.8
KZN-C2	21																					100	100
WC-10270	22																						100

Accession numbers of *BbSBP-4* sequences extracted from GenBank are (01 through 12): AB569303, AB569301, AB569300, AB594813, AB569302, AB617639, AB617641, AB571871, AB586125, AB594814, AF486506 and AF486507, respectively. Accession numbers of sequences generated in this study are (13 through 22): KF626632, KF626638, KF626636, KF626631, KF626629, KF626637, KF626634, KF626635, KF626630, KF626633

A multiple sequence alignment of *BbβTUB* sequences revealed the presence of SNPs among the South African *B. bovis* isolates and GenBank strains. The *BbβTUB* sequence from MP-C17 sample showed 97% identity with corresponding sequences from Texas (AK440534) and Samford (L00978) strains. MP-C17 sequence was also compared to other *BbβTUB* sequences determined in this study and the highest sequence identities recorded were between 97.2 and 97.5%. Sequences derived from other South African bovine samples (GP-C7, GP-C17, KZN-C2, INDIA, MP-C18, NW-C2, NW-C4 and WC-10270) showed 99% sequence identity when compared to those of Texas (AK440534) and Samford (L00978) strains.

### Phylogenetic tree analyses

**Fig. 2:**
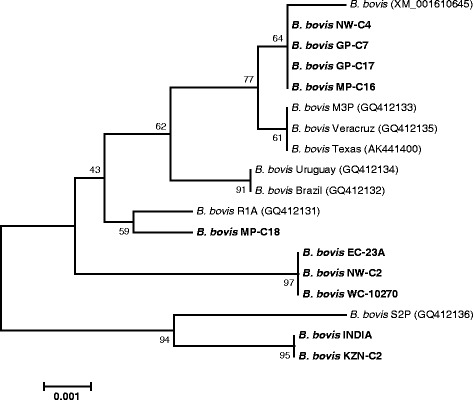
Phylogenetic tree inferred with *BbCP2* gene sequences (809 *nt*) of several *B. bovis* isolates from bovine samples collected at various geographical regions in South Africa, together with corresponding sequences retrieved from GenBank. Accession numbers are given in parentheses. Bootstrap values calculated as percentages of 1000 replicates are indicated at branching points. The horizontal scale bar shows the number of base substitutions per site

In order to study the phylogenetic relationship among the sequences of tested *B. bovis* isolates, nested PCR-amplified fragments of selected samples were sequenced in both strands. A neighbour-joining tree constructed with the *BbCP2* sequences showed a clear phylogenetic separation of sequences derived from geographical isolates of *B. bovis* (Fig. 2). The *BbCP2* sequences determined in this study were found in four different clusters. The first cluster grouped the reference strain (XM_001610645) with sequences from NW-C4, GP-C7, GP-C17 and MP-C16. Sequences of EC-23A, NW-C2 and WC-10270 formed a cluster clearly distinct from that of other sequences, with a high bootstrap support of 97%. While MP-C18 sequence clustered with that of R1A strain (GQ412131), the *BbCP2* sequences of INDIA and KZN-C2 exhibited a close phylogenetic relationship with the sequence of S2P strain (GQ412136).

**Fig. 3:**
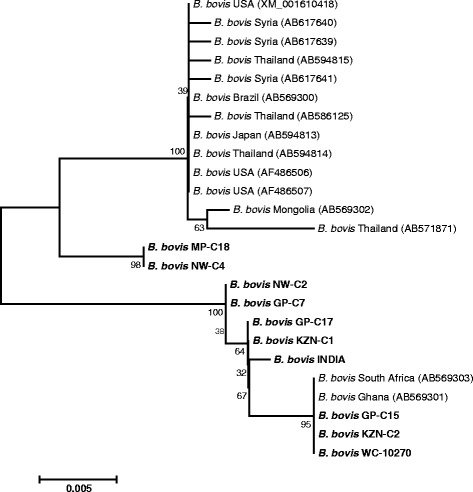
A neighbour-joining tree generated with *BbSBP-4* nucleotide gene sequences (726–744 *nt*) of South African *B. bovis* isolates (in bold type), together with sequences previously published in GenBank. Accession numbers are given in parentheses. Bootstrap values calculated as percentages of 1000 replicates are indicated at branching points. The horizontal scale bar shows the number of base substitutions per site

A phylogram created with neighbour-joining method based on *BbSBP-4* nucleotide gene sequences is shown in Fig. 3. The sequences obtained in this study fell into three clades that resulted from the existence of SNPs across the lengths of *BbSBP-4* genes. The sequences of MP-C18 and NW-C4 were clearly distinct from other sequences. The *BbSBP-4* gene sequences from GP-C7, GP-C17, KZN-C1, INDIA and NW-C2 all formed a separate grouping. Similarly, GP-C15, WC-10270 and KZN-C2 sequences showed a very close phylogenetic relationship with sequences of *B. bovis* strains published in GenBank (AB569301 and AB569303), and were supported by a high bootstrap value.

**Fig. 4:**
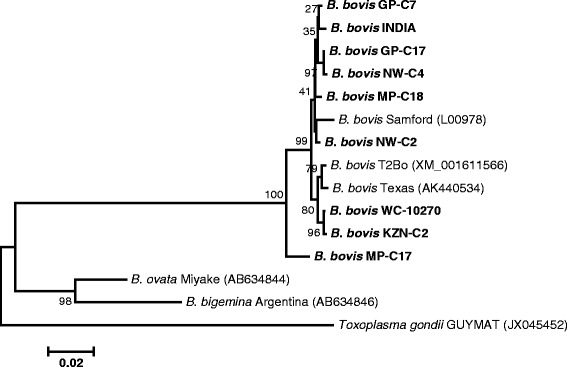
Phylogenetic tree based on *BbβTUB* nucleotide gene sequences (920 *nt*) derived from blood samples collected from South African cattle (in bold type), together with sequences previously published in GenBank (accession numbers in parentheses). Bootstrap values calculated as percentages of 1000 replicates are indicated at branching points. The horizontal scale bar shows the number of base substitutions per site. *Toxoplasma gondii* GUYMAT (accession no. JX045452) was used as an outgroup

The phylogenetic tree inferred with *BbβTUB* sequences is reflected in Fig. 4. In the phylogeny, it was observed that the sequences from GP-C7, GP-C17, INDIA, MP-C18, NW-C2 and NW-C4 clustered together. The DNA sequences derived from KZN-C2 and WC-10270 showed a grouping with Texas (AK440534) and T2Bo (XM_001611566) strains. Furthermore, it was noted that the *BbβTUB* sequence of MP-C17 isolate formed a separate cluster clearly distinct from that of other South African isolates, with a bootstrap support of 100%. Phylogenetic analysis of *BbβTUB* gene also indicated that *Babesia ovata* (AB634844) is genetically related to *B. bigemina* Argentina strain (AB634846).

## Discussion

The search for *B. bovis* vaccine that could confer absolute protective immunity against bovine babesiosis has been the subject of many studies over the last decades. To date, no recombinant subunit vaccines are available to eliminate babesial infections in cattle globally because of extensive antigenic variations displayed by heterologous parasitic strains. As a result, many research groups have intensified their efforts in an endeavour to search for novel vaccine candidates with potential to offer complete protective immune response against a challenge by heterologous strains.

In particular, many studies have focused on functionally important proteins that are believed to play a pivotal role in parasite survival and growth [17, 18]. For example, the RAP-1 protein is involved in the process of invasion of bovine red blood cells by merozoites [19, 20]. Peptidases, such as cysteine peptidase 2, possess enzymatic activities identified as virulence factors for Apicomplexan parasites [21]. The BbSBP is involved in stabilizing the environment after parasite invasion and plays a role in parasite growth [22]. In addition, BbSBP has been documented as a new serological antigen for global epidemiological research [23]. Likewise, the β-tubulin gene of *Babesia* parasites has been used as an informative marker for species discrimination [24].

The fight against bovine babesiosis in southern Africa has always relied on the use of live attenuated vaccines which are more prone to cross-contamination [6]. Furthermore, the practical application of these vaccines is hampered by genetic heterogeneities observed among field isolates [25]. Therefore, it remains imperative to document the genetic profiles of *B. bovis* parasitic field isolates in an endeavour to develop effective control strategies with potential to curtail infection of susceptible bovines by *Babesia* parasites.

In the present study, we describe the successful development and application of three nested PCR assays for the specific detection of *B. bovis* DNA in field bovine samples. The *BbRAP-1* nested PCR assay developed previously [12] was employed as the control for the newly developed nested PCR assays. Due to the high number of samples tested positive with *BbRAP-1* nested PCR-based assay, it appears that this assay was more sensitive than *BbCP2*, *BbSBP-4* and *BbβTUB* assays developed in the present study. However, it is reported in the literature that the high number of positive samples detected by *BbRAP-1* nested PCR may be attributed to the presence of several nearly identical copies of *B. bovis BbRAP-1* genes in the genome [26, 27].

To corroborate the specificities of nested PCR assays, PCR-generated amplicons from randomly selected positive samples were sequenced. Sequence analysis revealed high genetic conservation among the *BbRAP-1* DNA fragments of South African *B. bovis* isolates and those published in GenBank, with identities ranging between 99 and 100%. The high conservation of *BbRAP-1* genes in *B. bovis* field isolates is comparable to previous studies that observed significant similarities in *BbRAP-1* sequences of isolates from countries other than South Africa [10, 28].

Accordingly, in silico analysis of *BbCP2* sequences indicated a high degree of sequence conservation between South African *B. bovis* isolates and GenBank strains. In the BLAST search for homologous sequences in GenBank, the highest *BbCP2* sequence identities (99%) were recorded with *B. bovis* strains originating from Argentina, Mexico, Uruguay and Brazil. This suggested high conservation of *BbCP2* gene sequences among *B. bovis* strains from geographically distinct regions of the world. Although cysteine peptidases of *B. bovis* have not been well characterized [29], the evidence of the importance of peptidases for parasite growth and survival was obtained when specific inhibitors of these enzymes impaired merozoite growth in vitro [30]. Recent findings on in vitro and in vivo expression of bovipain-2, a cysteine protease, in *B. bovis* also suggest that peptidases might potentially be considered as important targets for the development of effective control strategies against bovine babesiosis [29].

Sequencing of *BbSBP-4* genes from *B. bovis* isolates examined in this study demonstrated the existence of gaps in some isolates, a feature commensurate with base-pair insertion or deletion events. These insertions and deletions of nucleotide bases within the open reading frames (ORFs) may result in frame shift mutations, depending on whether the nucleotides that are added to (insertion event) or deleted from (deletion event) the ORF are a multiple of three or not. To further investigate whether there were any significant differences between the protein sequences as well as the possibility of frame shift mutations among *B. bovis* isolates possessing gaps, the determined nucleotide sequences were translated to protein sequences in silico and aligned with corresponding sequences of *B. bovis* strains published in GenBank. Analysis of aligned protein sequences revealed high conservation between sequences, albeit there were amino acid differences observed along the stretch of the protein sequences. The conservation of BbSBP sequences among geographical isolates of *B. bovis* has also been observed elsewhere [31, 32].

In studying the phylogenetic relationship among *B. bovis* isolates based on *BbCP2*, *BbSBP-4* and *BbβTUB* gene sequences, neighbour-joining trees were inferred. The analyses of phylogenies indicated a close phylogenetic relationship between the South African *B. bovis* isolates and geographical strains originating from cattle in other countries, albeit the sequences showed varying phylogenetic groupings because of the existence of SNPs across the lengths of nucleotide gene sequences. However, the observed clustering of *BbCP2* and *BbSBP-4* sequences incorporated in the phylogenies should be interpreted with caution given that the trees were not rooted. Furthermore, the current dearth of *B. bovis BbβTUB* sequences in GenBank could not allow for a better discrimination of the phylogenetic relatedness between *B. bovis* isolates of South African origin and those of countries other than South Africa. Given the importance of β-tubulin protein-encoding gene as the molecular marker for species identification and discrimination [24], it is of paramount importance that more *BbβTUB* genes of geographical *B. bovis* strains are sequenced and made available in GenBank.

## Conclusion

In this study, we have described the development and application of three newly developed nested PCR assays for the specific detection and genetic characterization of *B. bovis* isolates in field bovine samples. Although only few sequences were incorporated in the phylogenetic analyses, it must be acknowledged that the findings from this study provide valuable information regarding the genetic conservation among *B. bovis* isolates of South African origin in comparison to those previously published in GenBank. Taken together, the data presented in this study has given an indication that immunologically important proteins encoded by the genes examined in this study could potentially be considered for exploitation as viable candidates for inclusion in recombinant subunit vaccines.
